# Intrinsic Dielectric Loss in Zr_0.8_Sn_0.2_TiO_4_ Ceramics Investigated by Terahertz Time Domain Spectroscopy

**DOI:** 10.3390/ma14010216

**Published:** 2021-01-05

**Authors:** Liviu Nedelcu, Cezar Dragos Geambasu, Monica Enculescu, Marian Gabriel Banciu

**Affiliations:** National Institute of Materials Physics, Atomistilor 405A, 077125 Magurele, Romania; cezar.geambasu@infim.ro (C.D.G.); mdatcu@infim.ro (M.E.)

**Keywords:** electroceramics, microwave dielectrics, zirconium tin titanate, dielectric resonators, intrinsic loss, terahertz time-domain spectroscopy

## Abstract

Terahertz time-domain spectroscopy (THz-TDS) was employed for estimation of intrinsic dielectric loss of Zr_0.8_Sn_0.2_TiO_4_ (ZST) ceramics. Single-phase ZST dielectric resonators (DRs) with various synthesis parameters and, consequently, different extrinsic losses, were prepared by conventional ceramic technology. Even though the DRs exhibit a similar microstructure, their quality factor (*Q* is the inverse of dielectric loss tangent) measured in microwave (MW) domain at 6 GHz varies between 2500 and 8400. On the other hand, it was found that the THz dielectric loss is less sensitive to the sample preparation. The intrinsic losses (*Q* × *f* ~60 THz) of the ZST ceramics have been derived from THz data.

## 1. Introduction

Microwave (MW) dielectrics continue to play a decisive role in the progress of information technology and communications, having an important contribution in downsizing and reliability improvement of the equipment. These materials were successfully used in passive devices like oscillators, multiplexers, filters, metamaterials, antennas, etc. [1,2,3,4,5,6,7,8,9]. Even though a large number of dielectrics with suitable properties have been reported [10,11,12,13,14,15], their mass production is restricted due to the manufacturing costs and technological limitations. As a consequence, ceramic materials became the most employed in passive MW devices due to the simplicity, cost effectiveness, and scalability of their large-scale fabrication.

Zr_1-x_Sn_x_TiO_4_ solid solutions [16] are very attractive for MW applications, and a composition with *x* = 0.2 offers a great temperature stability, being extensively investigated for dielectric resonator (DR)-based devices [1,2,13]. Such materials are generally difficult to be densified and their sinterability increases with appropriate additions. However, large amounts of dopants can degrade the real part ($\varepsilon^{\prime }$) and imaginary part ($\varepsilon^{\prime \prime }$) of the complex relative permittivity ($\varepsilon =\varepsilon^{\prime }-j\varepsilon^{\prime \prime }$) [13]. Dielectric loss is conventionally described by the dielectric loss tangent tanδ=ε″ε′ or by $\varepsilon^{\prime \prime }$. In the terahertz range, the dielectric loss is commonly described by the absorption coefficient. According to the multiphonon absorption theory, the *Q* × *f* product is constant in MWs and millimeter-waves (MMWs) for a defect-free material [17]. For this reason, the *Q* × *f* product is more frequently used as parameter of DRs rather than $\varepsilon^{\prime \prime }$ or *Q*, which vary with frequency. Zr_0.8_Sn_0.2_TiO_4_ (ZST) ceramics were fabricated by various methods and, analyzing the data published in the literature, it is worth mentioning that $\varepsilon^{\prime }$ vary between 35 and 40 while *Q* × *f* product is ranging from 10 THz to 100 THz [13,17,18,19,20,21,22,23].

In spite of the benefits mentioned above, the use of ceramics in MW applications can be limited due to their extrinsic dielectric loss. This drawback became particularly restrictive in the case of frequency-selective devices. Various sources of losses occur in dielectrics, and these are grouped in two classes: (i) Intrinsic [24], corresponding to electromagnetic field interaction with phonons in the ideal crystal, and (ii) extrinsic, related to such defects as secondary phases, grain boundaries, lattice local or extended defects, etc. [17]. Fortunately, the total (intrinsic + extrinsic) losses of polycrystalline materials can be reduced to the limit of the intrinsic ones by appropriate tuning of the “synthesis-microstructure-properties” cycle. Therefore, determination of the intrinsic losses is very important to decide when an optimization process should be stopped.

Over the time, many attempts have been made for estimation of the intrinsic losses of many MW dielectrics by using Fourier Transform Infrared (FTIR), Backward Wave Oscillator (BWO), and Whispering Gallery Mode (WGM) resonator techniques [10,13,25]. Of these, FTIR was the most employed because it enables the identification of the polar phonons which contribute to $\varepsilon^{\prime }$ and $\varepsilon^{\prime \prime }\;$in MWs and MMWs; the anharmonic interaction between the electromagnetic field and lattice vibrations is the dominant mechanism for losses, these being attributed to the damping of phonon modes. Based on this supposition, the extrapolation of losses to MWs and MMWs was made by using the proportionality between $\varepsilon^{\prime \prime }\;$and frequency [10,13].

Terahertz time-domain spectroscopy (THz-TDS) is an appropriate technique that allows us to measure the low-loss dielectrics in MMWs and submillimeter-waves thanks to its sensitivity, wideband capabilities, and ease of execution. Compared to other methods, THz-TDS has the advantage of a better signal-to-noise ratio than the BWO or FTIR [26]. Moreover, this spectroscopic technique provides both spectral intensity and phase shifts of the propagating field, which allow the direct determination of $\varepsilon^{\prime }$ and $\varepsilon^{\prime \prime }$ without using the Kramers–Kronig relations [27].

As a result of our previous investigations, ZST DRs with *Q* × *f* ~50 THz from MWs to MMWs were achieved by conventional ceramic technology [18]. The aim of this work is to exploit the versatility of the THz-TDS technique in order to determine the intrinsic dielectric loss of ZST ceramics. Three batches of DRs with different extrinsic losses were fabricated, as follows: Similar to the optimal condition (batch A) previously determined [18], close to optimal condition (batch B), and far from optimal condition (batch C); the MW and THz dielectric properties were systematically compared and discussed, while the intrinsic dielectric loss (*Q* × *f*) was estimated from THz data.

## 2. Materials and Methods

### 2.1. Samples Preparation

The Zr_0.8_Sn_0.2_TiO_4_ ceramics were prepared by conventional ceramic technology from high purity powders ZrO_2_ (Alfa Aesar, 99.5% purity, Kalrsruhe, Germany), SnO_2_ (Alfa Aesar, 99.9% purity, Kandel, Germany), and TiO_2_ (Sigma-Aldrich, 99.8% purity, Steinheim, Germany). In order to reduce the sintering temperature, 1 wt.% ZnO (Sigma-Aldrich, 99.0% purity, Steinheim, Germany) and 2 wt.% La_2_O_3_ (Sigma-Aldrich, 99.9% purity, Steinheim, Germany) were added. The powders were homogenized in the distilled water for 6 h in a PM200 planetary mill (Retsch, Haan, Germany) using zirconia balls and jars, and subsequently calcined in air at 1150 °C for 2 h. In order to avoid the Sn segregation on the grain boundaries during the sintering process, the calcined powders were mixed with 0.2 wt.% NiO (Sigma-Aldrich, 99.8% purity, Steinheim, Germany) and milled again for 2 h. The granulated powders were uniaxial pressed into cylindrical die to about 65% of ZST bulk density, 2 wt.% of polyvinyl alcohol being used as binder. Cylinders of 10 mm diameter and 5.5 mm height were sintered at 1300 or 1350 °C for 2–4 h with heating and cooling rates of 300 °C/h.

### 2.2. Experimental Details

The bulk density of the sintered samples was measured in distilled water by the Archimedes’ method with a Density Determination Kit (YDK01MS) mounted on a CUBIS MSA224S analytical balance (Sartorius Lab Instruments GmbH, Goettingen, Germany).

The structure of ZST samples was investigated by using X-ray diffraction (XRD) by employing a Bruker-D8 Advance diffractometer (Bruker AXS GmBH, Karlsruhe, Germany), in Bragg-Brentano geometry, equipped with a copper X-ray tube and LynxEye one-dimensional detector. A corundum plate NIST reference material (NIST SRM 1976) was used for checking the 2θ calibration of the instrument.

The morphology of the sintered samples was investigated using a Gemini 500 Field Emission Scanning Electron Microscope (Carl Zeiss, Oberkochen, Germany), working in both High Vacuum and Variable Pressure mode, from 0.2 to 30 kV, equipped with LaB6 filament, InLens, and SE2 detectors.

The sintered cylinders were employed as DRs and characterized in MWs with the TE_01δ_ Mode Dielectric Resonator (QWED, Warsaw, Poland) technique. The cavity was connected to an E8361A Vector Network Analyzer (Agilent, Santa Clara, CA, USA) and dielectric parameters where determined from transmission (*S*_21_ parameter) [28].

The terahertz measurements were carried out with an IRS 2000 PRO spectrometer (Aispec, Tokyo, Japan) on transmission set-up. Disks with thicknesses of about 0.5 mm were sliced from ZST resonators and the absorption coefficient, $\varepsilon^{\prime }$ and $\varepsilon^{\prime \prime }$ have been extracted from time-domain data by using TeraLyzer commercial software (Menlo Systems GmbH, Martinsried, Germany). In order to avoid the absorption of THz waves by atmospheric water vapor, the pressure in the samples chamber was kept below 10 Pa during the measurements.

## 3. Results and Discussion

Following the sintering process, ZST ceramics with relative density higher than 96.5% of X-ray density were obtained for all the batches. For accurate characterizations, the sintered cylinders were polished in order to remove the superficial layer. The bulk density of the ZST samples obtained in 3 sintering conditions is shown in Table 1. All samples have a porosity lower than 3.5%, which is a good achievement for uniaxial pressing.

**Figure 1:**
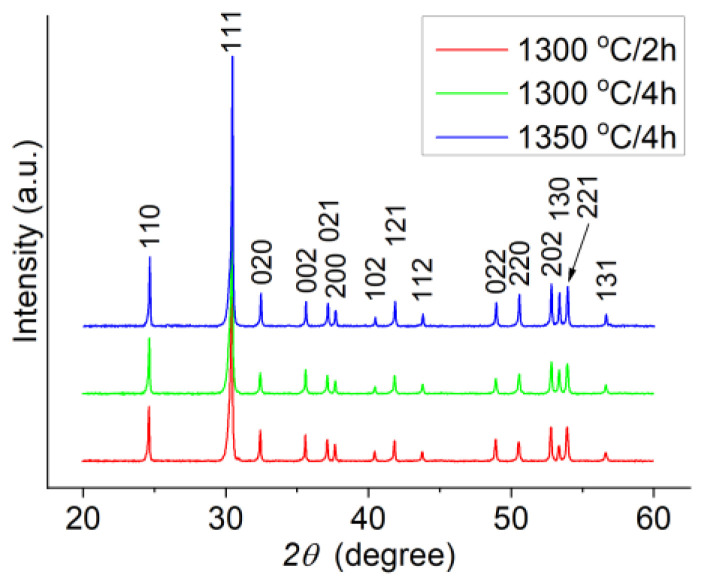
XRD (X-ray diffraction) patterns versus sintering condition for ZST ceramics.

The structural data obtained by XRD were analyzed using “Bruker Diffrac plus Basic Package Evaluation v.4.2.1” (Bruker AXS GmBH, Karlsruhe, Germany) and the phase composition was checked with ICDD PDF4+ 2020 database (International Centre for Diffraction Data, Newtown Square, PA 19073, USA). The XRD patterns presented in Figure 1 show the formation of the ZST composition with a αPbO_2_-type structure (space group Pnab). For all ZST batches, the diffraction lines were indexed with respect to the ICDD 01-081-2214 file and, within the detection limit of the diffractometer, no secondary phases were observed. The lattice constants *a, b*, and *c* of the orthorhombic unit cell extracted from the XRD patterns are listed in Table 1. As can be seen, the sintering conditions have a small influence on the lattice constants.

**Figure 2:**
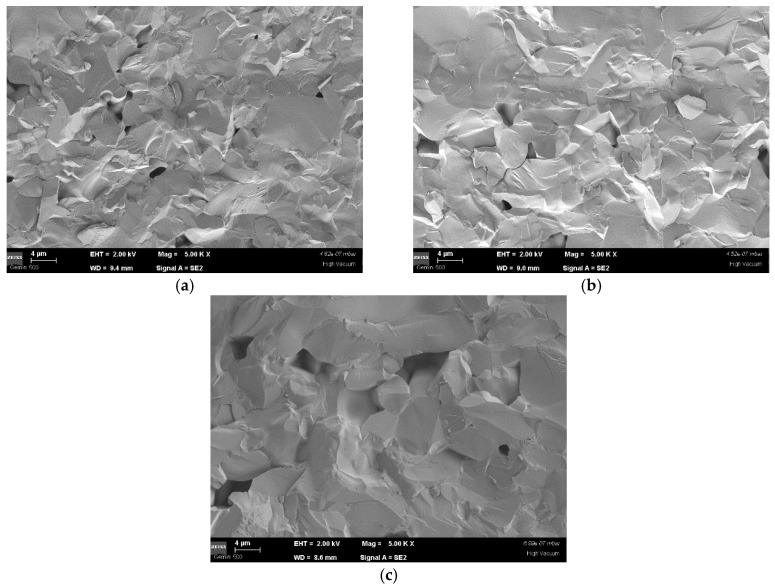
SEM (scanning electron microscopy) micrographs of the ZST samples sintered at (**a**) 1300 °C/2 h, (**b**) 1300 °C/4 h, and (**c**) 1350 °C/4 h. All the images were recorded using an accelerating voltage (EHT) of 2 kV, at working distances (WD) between 8.8 mm and 9.5 mm, and ×5000 magnification. The scale bars have 4 μm.

The microstructural characterization of the sintered ZST batches was performed by scanning electron microscopy (SEM). Figure 2 shows SEM micrographs of freshly cracked/broken/split surfaces of the sintered ceramics. Although at a first observation all the samples may look similar, with residual pores and grains of similar dimensions, several differences between the ZST batches may be underlined. Thus, the ZST samples obtained after 2 h of treatment at 1300 °C, whose SEM micrograph is presented in Figure 2a, is characterized by a significantly larger number of residual pores with relatively small dimensions. As the treatment time is increased, we may observe that a segregation of the pores is taking place, leading the presence of a smaller number of pores with larger dimensions in the sample, as observed in Figure 2b for the sample sintered at 1300 °C/4 h. Finally, an increase of the temperature during the treatment is causing a significant diffusion of the pores leading to the decrease of the pores’ number and the increasing of their volume, as observed for the ZST sample sintered at 1350 °C/4 h (Figure 2c). Taking into account the bulk density of the ZST ceramics (Table 1), the formation of those larger voids do not significantly change their porosity, the same volume of air being trapped between the grains of the sintered samples, but could affect their dielectric properties.

MW measurements of dielectric properties were conducted on ZST DRs with 8.8 mm diameter and 4.5 mm height. The effect of the sintering parameters on the complex relative permittivity and *Q* × *f* product of the A, B, and C batches is shown in Table 2. For comparison, their values extracted at 0.4 THz were added in Table 2 even though the THz-TDs investigation will be discussed later.

The obtained MW data revealed a small influence of the synthesis conditions on $\varepsilon^{\prime }$, which is consistent with densification data (Table 1) and SEM analysis (Figure 1). On the other hand, $\varepsilon^{\prime \prime }$ shows a very large variation, from 4.3 × 10^−3^ to 14.7 × 10^−3^. It is expected that the losses due to the pores do not vary too much for such reduced variation of the porosity (Table 1). Therefore, other extrinsic contributions are responsible for increased value of $\varepsilon^{\prime \prime }$ of ZST ceramics sintered close to optimal condition (batch B) and far from optimal condition (batch C).

As it was mentioned in the Introduction section, the *Q* × *f* is constant in MWs and MMWs for dielectric materials without defects. In practice, the measured value of the *Q* × *f* product increases with the increase of frequency [10,13,25,29], and this is a consequence of defects-related losses. On the other hand, its values reach a steady level in MMWs [25], which corresponds to intrinsic losses. In other words, the influence of defects on the total losses decreases with the increasing of frequency and, above a certain frequency, the extrinsic losses became negligible.

Even though the *Q* × *f* product is more or less dependent on frequency, it is used in most of the cases to describe the losses in microwave dielectric materials, being a catalog parameter of commercial manufacturers. Compared with DRs sintered at 1300 °C/2 h (batch A), the *Q* × *f* product given in Table 2 for ZST B and ZST C batches show significantly lower values. A noticeable result is that the increasing of the sintering time from 2 h (batch A) to 4 h (batch B) leads to a substantial increase of the extrinsic losses, an involution that could not be predicted from densification, structural, and morphological analyses.

**Figure 3:**
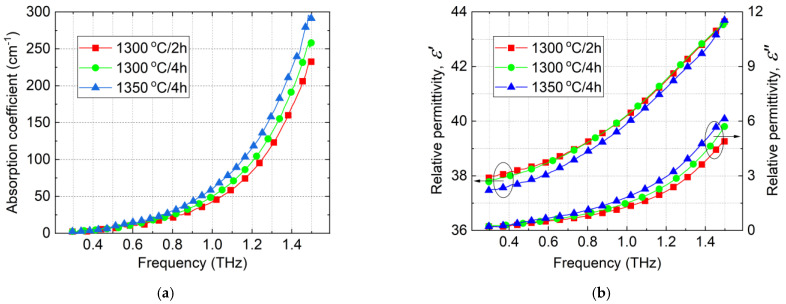
Frequency dependence of the absorption coefficient (**a**) and complex relative permittivity (**b**) versus sintering condition for ZST ceramics.

The frequency dispersion of the absorption coefficient, $\varepsilon^{\prime }$ and $\varepsilon^{\prime \prime }$ of ZST ceramics investigated by THz-TDS is given in Figure 3. Although the THz-TDS instrument is wideband and intrinsic silicon could be measured up to 7 THz, due to the ZST absorption, we could investigate the samples only up to 1.5 THz. Above this frequency, the transmitted signal is very weak.

The plots of dielectric parameters versus frequency depicted in Figure 3 show that the ZST A batch exhibited the lowest absorption coefficient and the lowest $\varepsilon^{\prime \prime }$ compared with the ZST B and ZST C batches. Moreover, the ZST A batch shows the highest dielectric permittivity compared to the other batches up to 0.8 THz. Even though the absorption coefficient and $\varepsilon^{\prime \prime }$ have variations from one batch to another, the contribution of extrinsic factors in THz is much lower than in MWs.

Comparing the dielectric properties of ZST batches in MWs with those from THz (Table 2), a small increase of $\varepsilon^{\prime }$ from 6 GHz to 0.4 THz is observed, which is in a reasonably agreement with the frequency invariant values predicted by the theory [10,17]. On the other hand, the *Q* × *f* product of ZST B and ZST C batches shows a significant increase from the values measured at MWs to the values measured at THz. However, the increase, which occurs for ZST A is much smaller. This is evidence of the fact that the extrinsic factors for ZST B and ZST C are more significant than for the best batch, ZST A.

From a general point of view, the THz-TDS is a very powerful technique for estimation of intrinsic dielectric loss of MW materials, especially in the case of polycrystalline ones. Unlike the MWs losses, the THz absorption is less sensitive to the sample preparation and, therefore, THz-TDS can be employed for tailoring of the synthesis parameter in an optimization cycle. Moreover, the THz-TDS gives $\varepsilon^{\prime }$ and $\varepsilon^{\prime \prime }$ without the need of additional modelling (computation), being very useful for fast screening of new dielectric materials. However, in order to have a complete and accurate image in the case of new materials, THz-TDS should be combined with at least one other spectroscopic technique (e.g., FTIR, BWO, WGMs) in MMWs or/and submillimeter-waves.

## 4. Conclusions

A systematic investigation of the extrinsic losses effect on the MW and THz dielectric properties of ZST ceramics is presented. Single-phase DRs with similar microstructure were fabricated by conventional ceramic technology. The measurements show that, while the extrinsic factor plays a significant role for losses in MWs, at such higher frequencies as terahertz frequencies the losses are given mainly by the intrinsic factor. The ZST A batch sintered at 1300 °C/2 h exhibited the lowest absorption coefficient and the highest $\varepsilon^{\prime }$ in the whole investigated frequency range, and their intrinsic dielectric loss (*Q* × *f* ~60 THz) was derived from THz data.

## Figures and Tables

**Table 1:** The bulk density and lattice constants of Zr_0.8_Sn_0.2_TiO_4_ (ZST) ceramics for different sintering conditions.

Batch	Sintering Temperature (°C)	Sintering Time (h)	Bulk Density ^1^ (g/cm^3^)	*a* (nm)	*b* (nm)	*c* (nm)
ZST A	1300	2	5.08	0.4774	0.5517	0.5044
ZST B	1300	4	5.05	0.4773	0.5516	0.5042
ZST C	1350	4	5.03	0.4772	0.5515	0.5041

^1^ The X-ray density of ZST is 5.193 g/cm^3^ (ICDD file no. 01-081-2214).

**Table 2:** Real part ($\varepsilon^{\prime }$) and imaginary part ($\varepsilon^{\prime \prime }$) of the complex relative permittivity and *Q* × *f* product of ZST ceramics measured in microwave (@ 6 GHz) and terahertz (@ 0.4 THz) domain.

Batch	$\varepsilon^{\prime }$ @ 6 GHz	$\varepsilon^{\prime \prime }$ @ 6 GHz	*Q* x *f* @ 6 GHz (THz)	$\varepsilon^{\prime }$ @ 0.4 THz	$\varepsilon^{\prime \prime }$ @ 0.4 THz	*Q* x *f* @ 0.4 THz (THz)
ZST A	37.3	4.3 × 10^−3^	50	38.2	2.5 × 10^−1^	60
ZST B	37.2	7.4 × 10^−3^	30	38.1	2.7 × 10^−1^	55
ZST C	37.1	14.7 × 10^−3^	15	37.6	2.9 × 10^−1^	50

## Data Availability

The data presented in this study are available on request from the corresponding author.
